# Torque Teno Virus in Kidney Transplant Recipients: Perspectives on Its Role as a Complementary Marker in Monitoring Net Immunosuppression

**DOI:** 10.3390/jcm15124682

**Published:** 2026-06-17

**Authors:** Patryk Wawrzonkowski, Jakub Mizera, Justyna Zachciał, Mirosław Banasik

**Affiliations:** 1 Department of Nephrology, Transplantation Medicine and Internal Diseases, Institute of Internal Diseases, Wroclaw Medical University, 50-551 Wroclaw, Poland; justyna.zachcial@umw.edu.pl (J.Z.); miroslaw.banasik@umw.edu.pl (M.B.); 2 University Clinical Hospital in Wroclaw, Borowska 213, 50-556 Wroclaw, Poland

**Keywords:** torque teno virus, TTV, kidney transplantation, immunosuppression monitoring, net immunosuppression, therapeutic drug monitoring

## Abstract

Monitoring immunosuppression in kidney transplant recipients remains challenging, as conventional therapeutic drug monitoring (TDM) reflects pharmacokinetic exposure rather than the overall functional immune state. Torque teno virus (TTV), a non-pathogenic virus, has emerged as a potential complementary biomarker of the net state of immunosuppression. This review evaluates the current evidence regarding the utility of TTV load in this context, focusing on its correlation with standard pharmacokinetic markers, the analytical performance of quantitative PCR assays, its role as an integrated marker of immunosuppression, and its predictive value for clinical outcomes. Available data indicate that TTV load shows weak and inconsistent correlations with individual drug levels, such as tacrolimus trough concentrations, supporting its role as a complementary rather than substitutive tool. qPCR-based assays demonstrate generally good sensitivity and reproducibility, although inter-assay variability and lack of standardization remain important limitations. Clinically, higher TTV levels have been associated with an increased risk of opportunistic infections, whereas lower levels have been linked to acute rejection, suggesting a potential association between TTV viremia and immune status. TTV monitoring may represent a promising complementary approach for a more individualized assessment of immunosuppression. However, further prospective and interventional studies are required to validate standardized thresholds and determine whether TTV-guided strategies improve transplant outcomes compared with conventional monitoring.

## 1. Introduction

Long-term graft function in kidney transplant recipients depends on maintaining an appropriate balance between preventing alloimmune injury and avoiding excessive immunosuppression [1]. This balance is primarily achieved through the administration of immunosuppressive agents, including calcineurin inhibitors, corticosteroids, and mycophenolate mofetil [2]. While these drugs are highly effective in promoting immunological tolerance and preventing graft rejection, they are usually characterized by a narrow therapeutic index. Consequently, inappropriate dosing can lead to subtherapeutic exposure or toxic adverse effects [2]. Additionally, the requirement for lifelong immunosuppressive therapy poses challenges to patient adherence and compliance, further complicating treatment outcomes [3]. Given these concerns, rigorous monitoring of immunosuppressive therapy is critical to ensure both the efficacy and safety of treatment in kidney transplant recipients.

Therapeutic drug monitoring (TDM) of immunosuppressive agents is an essential component of post-transplant care and is routinely applied, particularly in the case of calcineurin inhibitors such as tacrolimus [4]. Monitoring whole-blood tacrolimus trough concentrations remains a standard approach, as maintaining appropriate drug exposure is critical: subtherapeutic concentrations can lead to insufficient immunosuppression and increase the risk of immunological graft injury, whereas supratherapeutic concentrations are associated with significant drug-related nephrotoxicity [5,6]. However, this method is limited by substantial interindividual variability in drug metabolism, primarily mediated by cytochrome P450 3A4 and 3A5 (CYP3A4 and CYP3A5) enzymes in the liver and intestines. Moreover, the activity of CYP3A4 is influenced by numerous endogenous and exogenous factors, including enzyme inducers and inhibitors, which can result in unpredictable fluctuations in drug levels [7]. These limitations underscore the need for more precise and individualized approaches to TDM, capable of overcoming the challenges posed by current methods.

Emerging evidence suggests a potential role for Torque teno virus (TTV) in immunosuppression monitoring as a complementary marker that may support a more individualized assessment of kidney transplant recipients [8,9,10]. TTV is a nonpathogenic, ubiquitous single-stranded DNA virus whose replication is influenced by the host immune system [11]. For consistency, the abbreviation “TTV” is used after the first mention. In immunocompetent individuals, TTV load is generally low and stable; however, in states of immunodeficiency or more intense immunosuppression, viral titers may increase [12]. Several cohort studies in kidney transplant populations have suggested an inverse association between peripheral blood TTV load and immune competence. In these studies, higher TTV viremia was associated with markers of reduced lymphocyte-mediated immune function and an increased risk of opportunistic infections, whereas lower TTV levels were linked to rejection episodes or subclinical alloreactivity [8,9,12].

Quantitative PCR assays for TTV have shown promising analytical performance and may allow frequent, noninvasive assessment of TTV viremia in transplant recipients [13,14]. In contrast to tacrolimus TDM, which provides information on the pharmacokinetics of a single agent, TTV load has been proposed as a broader marker that may reflect the cumulative effects of immunosuppressive therapy, host-related factors, and immune competence [15]. This broader scope may make TTV monitoring potentially useful in complex immunosuppressive regimens, although its interpretation should remain integrated with conventional pharmacokinetic monitoring and clinical assessment. Rather than directly guiding immunosuppressive dose adjustments, TTV quantification may provide complementary information on the patient’s overall immunological status and may help identify individuals at increased risk of infection or rejection. However, its ability to improve graft outcomes or patient safety through TTV-guided therapeutic strategies remains to be confirmed in prospective interventional studies [15].

However, the clinical interpretation of TTV remains challenging. Existing studies have reported heterogeneous correlations between TTV load and individual immunosuppressive drug levels, variability between qPCR assays, lack of standardized thresholds, and limited prospective evidence supporting TTV-guided therapeutic decisions [10,13]. Therefore, although TTV appears to be a promising complementary biomarker of the net state of immunosuppression, its clinical utility remains insufficiently validated.

The aim of this review is to critically evaluate the current evidence regarding TTV as a complementary biomarker of net immunosuppression in kidney transplant recipients, focusing on its relationship with conventional pharmacokinetic monitoring, analytical performance of qPCR-based assays, proposed clinical thresholds, and the evidence gaps that must be addressed before TTV can be incorporated into routine immunosuppressive management.

## 2. Materials and Methods

This article was designed as a narrative literature review structured around predefined review questions. The review focused on evidence regarding the use of TTV as a complementary marker of net immunosuppression in kidney transplant recipients, with particular attention to pharmacokinetic monitoring, analytical assay performance, clinical outcome associations, and current limitations of implementation.

(A) RQ1: How does peripheral blood TTV load correlate with traditional tacrolimus trough concentrations and other pharmacokinetic markers of immunosuppression?
(B) RQ2: What are the analytical performance characteristics of quantitative PCR assays for measuring TTV in transplant patients?
(C) RQ3: To what extent may TTV load reflect aspects of the net state of immunosuppression—integrating multiple drug effects and individual host factors—compared with single-agent TDM?
(D) RQ4: How are proposed TTV load thresholds associated with clinically meaningful outcomes, such as opportunistic infections and acute rejection episodes, compared with conventional drug-level monitoring?

The review questions were conceptually structured using the PICO framework, which clarifies four essential elements—Population/Problem (P), Intervention/Exposure (I), Comparison (C), and Outcome (O)—to support a targeted narrative synthesis [16]. In this study, these components were defined as follows:

P—kidney transplant recipients.
I—TTV monitoring as a potential complementary marker of net immunosuppression.
C—conventional immunosuppression monitoring methods, including therapeutic drug monitoring.
O—associations with clinically relevant outcomes, including infection risk, rejection episodes, graft function, and adverse effects related to immunosuppressive therapy.

The literature review began with the identification of key information databases, including PubMed, MEDLINE, Scopus, and Google Scholar. The following combinations of search terms were used: “Torque teno virus” OR “TTV” OR “torque teno virus” combined with “kidney transplantation” OR “kidney transplant recipients” OR “renal transplantation” and “immunosuppression” OR “immune monitoring” OR “therapeutic drug monitoring” OR “tacrolimus” OR “mycophenolic acid” OR “infection” OR “rejection”. An example PubMed search string was (“Torque teno virus” OR “TTV” OR “torque teno virus”) AND (“kidney transplantation” OR “kidney transplant recipients” OR “renal transplantation”) AND (“immunosuppression” OR “immune monitoring” OR “therapeutic drug monitoring” OR “tacrolimus” OR “infection” OR “rejection”). Searches were adapted to the syntax of each database. In Google Scholar, simplified combinations of the same terms were used to identify additional relevant articles.

The literature search included peer-reviewed articles published in English or Polish and available up to August 2025. This cut-off date corresponded to the final structured literature update performed for the narrative synthesis; therefore, studies published after this date were not included. Eligible publications were full-text articles evaluating TTV viral load in relation to immunosuppression, immune status, infection risk, rejection, graft function, vaccine responsiveness, or other clinically relevant outcomes in kidney transplant recipients.

Titles and abstracts were screened to exclude clearly irrelevant reports, and potentially relevant articles were subsequently assessed in full text. Publications were evaluated for relevance to the predefined review questions, study design, sample size, analytical methods, timing of TTV measurement, reported clinical endpoints, and methodological transparency. Articles lacking sufficient methodological detail or falling outside the topic or date range were excluded. Data from the selected studies were organized to support a narrative synthesis and to identify major gaps in the literature.

To facilitate comprehension of the principal evidence discussed in this narrative review, Table 1 provides a structured summary of selected key articles included in the synthesis.

## 3. Correlation of Peripheral Blood TTV Load with Tacrolimus Levels and Pharmacokinetic Markers, and Its Evaluation as an Integrated Marker of Immunosuppression Beyond Single-Agent Monitoring

Therapeutic drug monitoring (TDM) of immunosuppressive agents is the most widely used strategy in current clinical practice to assess pharmacokinetic exposure after solid-organ transplantation [28,29]. This approach involves titrating drug dosages based on quantitative levels obtained from biofluids, most commonly whole blood or plasma. Currently, the immunosuppressive agents most frequently subjected to therapeutic drug monitoring in kidney transplantation are calcineurin inhibitors, including tacrolimus and cyclosporine, mycophenolic acid (MPA), and mTOR inhibitors, such as sirolimus and everolimus [30]. TDM is employed to individualize immunosuppressive therapy, minimize the risk of drug-related toxicity, and reduce the risk of graft rejection [31]. Nevertheless, despite its broad range of applications, TDM is associated with several important limitations.

Significant variability is frequently observed among results obtained from different commercially available assays. Data from proficiency testing—where laboratories analyze and report results for unknown samples—highlight considerable discrepancies between the various methods used to quantify the same drug. For instance, a single drug may be analyzed using more than half a dozen approaches, including different immunoassays, fluorescence polarization, or turbidimetric techniques, with multiple vendors providing each type of assay on diverse instrumentation platforms.

Another challenge in therapeutic drug monitoring arises from frequent preanalytical errors, particularly incorrect sample timing. This occurs when trough or peak levels are drawn at moments that do not correspond to the true minimum or maximum concentration of the drug. Mistimed sampling can also occur if samples are collected before the drug reaches steady-state concentrations. Proper timing depends on an understanding of the specific pharmacokinetics of each drug, including its route of administration and half-life [17].

Recent evidence suggests that TTV load may reflect aspects of the net state of immunosuppression—that is, the cumulative effects of immunosuppressive therapy, patient-specific factors, and biological variables—rather than showing a simple linear relationship with a single pharmacokinetic parameter. Higher TTV loads have been associated with an increased risk of infection and immunosuppression-associated adverse events, whereas lower loads have been linked to acute rejection episodes [18,19]. Thus, assessment of TTV viral load may help stratify the risk of infection or rejection, but current evidence does not support its use as a standalone indicator of over- or under-immunosuppression.

Correlations between TTV and tacrolimus trough levels (C0), as well as between TTV and other pharmacokinetic measures, such as mycophenolate concentrations or area under the curve (AUC), are variable: some cohorts show a weak positive correlation, whereas others demonstrate no significant association. Therefore, TTV appears to serve as a complementary, rather than substitutive, marker relative to conventional drug-level measurements [10,20].

Several observational and review studies report a weak or absent correlation between current tacrolimus trough concentrations (C0) and contemporaneous TTV levels. For example, systematic analyses and cohort studies suggest that TTV may provide complementary risk-stratification information for infection or rejection beyond CNI trough levels alone [21]. However, some studies, such as clinical cohort reports and pre/post-immunosuppression regimen analyses, have demonstrated a modest positive correlation between rising TTV levels and higher tacrolimus concentrations or an increased overall immunosuppressive burden. For example, Bredewold et al. reported a correlation between TTV load and tacrolimus trough levels (C0) in their cohort [21].

For mycophenolate (MPA) and MPA–AUC, multiple studies have not demonstrated a consistent correlation with TTV levels; some analyses report no association, while others show only an indirect relationship in the context of dose adjustments. Similarly, for cyclosporine and mTOR inhibitors, findings are heterogeneous, and review articles emphasize that TTV does not reliably reflect the instantaneous concentrations of these agents [10,32].

The causes of this heterogeneity, as well as the biological mechanisms underlying these discrepancies, remain incompletely understood and likely reflect differences in study design, patient populations, immunosuppressive regimens, and the multifactorial nature of TTV dynamics. As noted above, TTV has been proposed to reflect aspects of the net state of immunosuppression, rather than pharmacokinetic drug exposure alone. Because TTV replicates under conditions of reduced immune competence, its viral load may depend on a broad spectrum of factors, including pharmacokinetics, such as drug dose and blood levels; pharmacodynamics, such as the drug’s effect on lymphocyte function; patient adherence; coexisting inflammatory or infectious conditions; age; frailty; and host genetics. Consequently, a single, point-in-time tacrolimus trough level may be insufficient to capture this biological complexity [22].

Changes in TTV load occur with a substantial delay, on the order of several weeks. Interventional studies and protocol-based analyses indicate that meaningful alterations in immunosuppression may become reflected in TTV levels only after approximately 6–12 weeks. Therefore, attempts to correlate a single, contemporaneous tacrolimus trough concentration (C0) with TTV at the same time point are inherently limited. This delay helps explain why correlations with short-term tacrolimus fluctuations are typically weak [23,24].

From a clinical perspective, these findings raise the question of how TTV could be integrated with pharmacokinetic monitoring to improve immunosuppression assessment. TTV may be considered a clinically contextualized, complementary tool to pharmacokinetic measurements, such as C0 and AUC. Drug-level monitoring remains essential for assessing toxicity and interactions, while TTV may provide additional information on the risk of infection or rejection arising from the overall immunosuppressive effect [20]. Serial TTV measurements and trend analysis may be more informative than isolated measurements, as rapid therapeutic decisions based solely on a single TTV value may be unreliable due to the virus’s kinetics [23].

The clinical context is also an important consideration. High TTV levels combined with stable, “within-range” tacrolimus C0 may suggest a higher overall immunosuppressive burden or greater pharmacodynamic sensitivity, whereas low TTV despite high C0 may reflect preserved immune competence, biological variability, or a recent dose increase that has not yet affected TTV levels [33,34].

Taken together, these findings highlight several evidence limitations and research gaps. In this context, there is considerable heterogeneity in TTV assays, including different qPCR methods and commercial kits, as well as a lack of standardized interpretive thresholds. Small cohort sizes, varying post-transplant sampling times, and diverse immunosuppressive regimens further complicate comparisons across studies. Prospective interventional studies are needed to determine whether therapeutic strategies incorporating TTV monitoring, in addition to standard C0-guided management, improve outcomes such as infection, rejection, and graft function [23].

Moreover, further studies are needed to evaluate how individual immunosuppressive agents influence TTV viral load. Given that kidney transplant recipients are generally maintained on standard triple immunosuppressive therapy, such investigations may clarify the relationship between specific drugs and TTV dynamics, thereby improving the understanding of how different immunosuppressive regimens contribute to the net state of immunosuppression [25].

## 4. The Analytical Performance Characteristics (Sensitivity, Specificity, and Reproducibility) of Quantitative PCR Assays for Measuring TTV in Transplant Patients

Quantitative real-time PCR (qPCR)-based assays currently constitute the primary method for measuring Torque teno virus (TTV) viremia in studies focused on immunosuppression monitoring after kidney transplantation [13]. The literature describes both in-house assays and commercial kits, as well as adaptations for automated platforms; these methods differ in analytical sensitivity, including limit of detection (LoD), linearity, precision, intra- and inter-assay coefficient of variation (CV), genotypic coverage, and interlaboratory reproducibility [35].

Analytical performance is discussed according to key parameters reported in validation and method-comparison studies, including limit of detection, linearity, precision, genotypic coverage, sample matrix, calibration approach, and interlaboratory reproducibility. Because assay design, calibration procedures, sample matrices, and reporting standards vary across studies, the available data do not allow a formal comparative ranking of all TTV qPCR platforms.

Published qPCR assays demonstrate limits of detection in the range of several tens to a few hundred copies per mL, often also expressed as log10 copies/mL. For example, the assay described by Kulifaj et al. demonstrated a limit of detection of approximately 2.2 log10 copies/mL, corresponding to approximately 160 copies/mL, in both whole blood and plasma, with confirmed linearity and precision across a dynamic range of approximately 1.61–10.61 log10 copies/mL [13]. Similarly, the automated adaptation on the Hologic Panther Fusion platform described by Spezia et al. demonstrated a limit of detection of approximately 1.6 log10 copies/mL, corresponding to approximately 42 copies/mL at a 95% hit rate, and the ability to detect single replicates at concentrations of approximately 85 copies/mL [25]. Accordingly, inter-assay differences in limits of detection, on the order of tens versus hundreds of copies/mL, may influence the classification and interpretation of low-level viremia, particularly when TTV values are close to proposed interpretive cut-offs. These differences are particularly relevant when comparing studies that use different analytical platforms or assay calibration procedures. Therefore, meaningful cross-study comparisons and interpretation of TTV measurements require reporting both absolute viral loads, expressed as copies/mL, and log10-transformed values, together with explicit specification of the assay used and its corresponding limit of detection.

Regarding assay precision, coefficient of variation data are available from validation studies. Spezia et al. reported intra-run CVs ranging from 1.47% to 2% for control levels around 4.0 log copies/mL and an inter-run CV of approximately 1.7%, indicating good reproducibility of the automated method [25]. Validation studies of both commercial kits and in-house assays typically report intra-assay and inter-assay CVs within a few percent, depending on viral load levels, although higher variability is generally observed at concentrations close to the limit of detection [35]. Low coefficients of variation, particularly within the mid-range of quantification, may provide sufficient analytical confidence for interpreting relative changes in serial TTV measurements, for example, increases of ≥0.5–1.0 log10. However, measurements near the limit of detection should be interpreted with greater analytical uncertainty, because small changes in viral load may fall within assay-related variability. These parameters are therefore important for distinguishing analytically meaningful changes from variability related to the assay itself and for comparing results across studies [13,25].

Most routine qPCR assays target the highly conserved untranslated region (UTR) of TTV. Described assays generally demonstrate the ability to detect a broad spectrum of TTV genotypes and show no cross-reactivity with other viruses commonly present in blood. Kulifaj et al. reported that their assay detects the most prevalent human TTV genotypes and does not cross-react with any of the viruses tested during validation [13]. Nevertheless, anelloviruses are genetically heterogeneous, and some primer/probe designs may vary in their ability to detect rare strains. Consequently, inter-assay or interlaboratory comparisons may show minor discordances, particularly at low viremia levels. Method comparison studies, including head-to-head evaluations, have generally demonstrated high concordance, although agreement is not complete near the limit of detection [36].

Standardization remains essential for comparing results across studies, particularly in multicenter investigations and analyses evaluating proposed viral load cut-offs. Some commercial assays provide the option to convert copies/mL to international units per mL (IU/mL) relative to the 1st World Health Organization International Standard, which may improve comparability [37]. However, the availability of such calibration does not fully eliminate differences related to sample matrix, extraction procedures, primer/probe design, and platform-specific analytical performance; therefore, TTV values obtained using different assays or platforms may not be directly interchangeable.

When discussing TTV measurement methods, pre-analytical factors that may influence results should also be considered. The sample matrix, including whole blood, plasma, or serum, can influence reported viral loads, as some assays are validated for a specific matrix. These differences may result in systematic discrepancies in copies/mL between studies and may affect whether a given TTV value is interpreted as low, intermediate, or high in relation to proposed cut-offs; they should therefore be carefully considered when interpreting and comparing results across different investigations [13]. Pre-analytical and analytical variables, including the extraction procedure, extraction platform, input volume, the presence of PCR inhibitors, and differences in assay calibration, may significantly affect the limit of detection and assay reproducibility. The use of automated systems may reduce operator-related variability, thereby enhancing the consistency and reliability of the measurements [35].

In summary, qPCR-based assays for TTV generally demonstrate adequate analytical sensitivity and precision for quantifying changes in viral load within the validated dynamic range of each assay. However, these analytical characteristics directly influence the interpretation of TTV measurements: results near the limit of detection carry greater uncertainty, small viral-load changes may fall within assay variability, and values obtained using different matrices or platforms may not be directly comparable [13,36]. The lack of full harmonization, including differences in assays, limits of detection, calibration procedures, and sample matrices, limits direct comparability between studies and complicates the use of uniform interpretive cut-offs. Therefore, assay-specific validation parameters and the criteria used for assay selection should be clearly reported whenever TTV viral load data are presented, and published cut-offs should not yet be treated as universally applicable across different analytical platforms [37,38].

## 5. Proposed TTV Load Thresholds and Their Association with Clinical Outcomes Compared with Conventional Drug-Level Monitoring

Cohort studies have reported an association between higher TTV viremia levels and an increased risk of opportunistic infections after kidney transplantation; however, the strength and reproducibility of these associations vary across studies. For instance, patients who developed infections exhibited higher mean TTV loads, 5.39 log10 versus 4.16 log10 copies/mL. In this cohort, an optimal predictive threshold of approximately 5.16 log10 copies/mL yielded a sensitivity of approximately 60% and a specificity of approximately 81% for infection risk classification, while the application of a machine-learning model improved sensitivity to approximately 80% while maintaining good specificity [39]. These findings suggest potential risk-stratification value, but they should be interpreted in the context of cohort-specific methodology, endpoint definitions, and statistical modeling.

In another prospective study, TTV levels measured at 1 and 3 months post-transplantation were associated with subsequent infection risk during later follow-up periods, with AUC-ROC values of approximately 0.72–0.78, despite the absence of a significant correlation with exposure to standard immunosuppressive drugs, such as tacrolimus or mycophenolic acid [26]. However, not all studies have confirmed consistent threshold effects, and reported cut-offs may vary depending on the assay used, sample matrix, timing after transplantation, immunosuppressive regimen, and clinical endpoint definition. In addition, meta-analyses indicate that lower TTV viremia is associated with a higher risk of rejection or subclinical alloreactivity, whereas higher TTV levels are linked to a reduced risk of rejection, supporting the concept of TTV as a potential bidirectional biomarker of the net state of immunosuppression [21].

Recent review articles have proposed specific TTV thresholds for post-transplant complication risk; however, these thresholds remain investigational. A proposed viremia range between 4.6 and 6.6 log10 copies/mL has been suggested to balance the risks of rejection and infection during months 4–12 post-transplant, with values above this range suggesting over-immunosuppression and increased infection risk, and values below this range indicating an elevated risk of rejection or subclinical alloreactivity [27]. Nevertheless, this range should be interpreted as hypothesis-generating rather than as a validated therapeutic target, particularly given the heterogeneity of available studies and the lack of standardized thresholds.

Conventional monitoring of immunosuppressive drug levels, including tacrolimus and MPA, measures pharmacokinetic exposure but does not always correlate directly with the functional state of the immune system or predict clinical outcomes, such as the risk of infections or rejection [40,41]. Importantly, available studies suggest that TTV viremia may help identify patients at increased risk of infection even when standard immunosuppressive parameters remain within the “therapeutic range”; in one study, TTV was associated with subsequent infections despite showing no correlation with tacrolimus or mycophenolic acid variability or exposure, supporting its potential role as a complementary functional biomarker of net immunosuppression [26]. However, these observations should not be interpreted as evidence that TTV thresholds are superior to conventional monitoring, as direct comparative validation remains limited.

Proposed TTV load thresholds have been associated with the risk of post-transplant complications, including infections and rejection, and may provide complementary information beyond conventional drug-level monitoring. However, existing evidence is primarily derived from observational cohorts and meta-analyses, and the apparent risk-stratification value of TTV thresholds may be influenced by publication bias, selective reporting of positive thresholds, differences in statistical modeling, and heterogeneity in assay methodology. Therefore, the comparative risk-stratification performance and clinical utility of TTV-guided monitoring require validation in prospective interventional studies.

## 6. Conclusions

This review highlights Torque teno virus (TTV) as a promising complementary biomarker of the net state of immunosuppression in kidney transplant recipients. Unlike conventional therapeutic drug monitoring, TTV load may reflect the combined effects of immunosuppressive therapy and host-related factors, while showing limited correlation with individual drug levels. Higher TTV levels have been associated with an increased risk of infection, whereas lower levels have been linked to allograft rejection, supporting its potential role as a functional marker of immune status. However, the predominance of observational data, heterogeneity of available studies, lack of assay standardization, and absence of validated thresholds currently limit its clinical applicability. Further prospective interventional studies are required to determine whether TTV-guided monitoring can improve clinical outcomes beyond conventional therapeutic drug monitoring. Notably, this question is currently being addressed in the randomized phase II TTVguideIT trial comparing standard and TTV-guided immunosuppression in kidney transplant recipients.

Beyond infection and rejection risk stratification, the broader clinical meaning of TTV dynamics remains incompletely defined. Emerging data suggest that TTV load may also be related to vaccine responsiveness in transplant recipients, particularly after SARS-CoV-2 vaccination, as higher TTV loads have been associated with weaker humoral and/or cellular vaccine responses in kidney transplant recipients [42,43]. TTV dynamics may also be relevant in the context of CMV reactivation, although available studies differ in design, population, and timing of sampling [18,37]. Moreover, other human anelloviruses, including torque teno mini virus (TTMV) and torque teno midi virus (TTMDV), may represent complementary markers of immune status, although their clinical value in transplantation remains insufficiently defined [44,45]. These potential applications, together with the economic feasibility and reimbursement of repeated viral load testing, require further evaluation before TTV-based monitoring can be considered for routine clinical implementation [27].

## Figures and Tables

**Table 1:** Summary of principal articles included in the narrative review.

Reference	Study Type/Context	Main Findings	Contribution to the Narrative Synthesis
Fernández-Ruiz, 2020 [8]	Expert commentary/conceptual perspective in solid organ transplantation	Discussed TTV load as a surrogate marker of the net state of immunosuppression and highlighted the potential clinical value of the anellovirus virome.	Provides the conceptual basis for interpreting TTV as a complementary immune-status marker rather than as a direct pharmacokinetic parameter.
Forqué et al., 2023 [9]	Prospective observational study in kidney transplant recipients	Evaluated dynamics of human anelloviruses in plasma and their relationship with post-transplant clinical outcomes.	Supports the relevance of longitudinal anellovirus/TTV kinetics for clinical risk stratification after kidney transplantation.
Kelly et al., 2024 [10]	Clinical study in pediatric kidney transplant recipients	Assessed TTV load as a marker of immunosuppression in a pediatric kidney transplant population.	Extends the discussion of TTV monitoring beyond adult cohorts and illustrates population-specific considerations.
Kulifaj et al., 2018 [13]	Analytical validation study of a standardized real-time PCR assay	Developed and validated a real-time PCR approach for TTV detection and quantification, including analytical sensitivity and dynamic range.	Supports the analytical feasibility of TTV viral-load monitoring and the need to report assay-specific validation parameters.
Görzer et al., 2023 [14]	Validation study using a CE-certified PCR assay in kidney transplantation	Validated plasma TTV load measurement for risk stratification of rejection and infection after kidney transplantation.	Provides evidence that standardized commercial assays may support clinical risk stratification, while harmonization remains necessary.
Strassl et al., 2019 [15]	Observational study focused on biopsy-proven alloreactivity	Reported an association between TTV load and acute biopsy-proven alloreactivity in kidney transplant recipients.	Supports the concept that low TTV levels may be linked to insufficient net immunosuppression and rejection risk.
Maggi et al., 2018 [17]	Prospective study in solid organ transplant recipients	Showed that early post-transplant TTV viremia was associated with later CMV reactivation.	Illustrates the potential relationship between TTV dynamics, global immune status, and viral infectious complications.
Strassl et al., 2018 [18]	Prospective biomarker study in kidney allograft recipients	Quantified TTV viremia as a prospective biomarker for infectious disease after kidney transplantation.	Supports the association between higher TTV loads and infection risk in kidney transplant recipients.
Reineke et al., 2024 [19]	Observational study in kidney transplant recipients with indication biopsy and therapeutic modifications	Evaluated TTV load dynamics in relation to clinically indicated biopsy findings and changes in immunosuppressive therapy.	Highlights the potential value of serial TTV measurements in clinically complex scenarios rather than relying on isolated values.
van Rijn et al., 2023 [20]	Systematic review and meta-analysis in solid organ transplantation	Synthesized evidence on TTV load as a marker of rejection and infection across transplant populations.	Provides higher-level evidence for TTV as a bidirectional biomarker while emphasizing between-study heterogeneity.
Bredewold et al., 2024 [21]	Cohort study evaluating immunosuppressive regimen effects	Assessed changes in TTV load in kidney transplant recipients treated with calcineurin inhibitors and after conversion to belatacept.	Supports the view that TTV may reflect the global biological effect of different immunosuppressive strategies rather than exposure to a single drug.
Haupenthal et al., 2023 [22]	Randomized controlled phase II trial protocol (TTVguideIT)	Described a multicenter trial comparing standard immunosuppression with TTV-guided immunosuppression in kidney transplant recipients.	Shows that the key clinical question of whether TTV-guided monitoring improves outcomes is being prospectively tested.
Querido et al., 2023 [23]	Prospective cohort study during the first post-transplant year	Reported associations between TTV viral-load kinetics, infection, and de novo donor-specific antibodies.	Supports the importance of TTV kinetics and repeated measurements for post-transplant immune monitoring.
van Rijn et al., 2021 [24]	Clinical cohort study in kidney transplantation	Found that TTV loads after kidney transplantation predicted allograft rejection but not viral infection.	Demonstrates that TTV performance may vary by endpoint and cohort, reinforcing the need for cautious interpretation.
Spezia et al., 2024 [25]	Analytical validation on a fully automated random-access platform	Developed real-time PCR TTV quantification on an automated platform with favorable reproducibility characteristics.	Supports the technical feasibility of repeated TTV testing and the potential reduction in operator-dependent variability.
Cañamero et al., 2023 [26]	Prospective observational study in kidney transplant recipients	Reported that TTV load predicted opportunistic infections but was not associated with maintenance immunosuppression exposure.	Supports the key conclusion that TTV may reflect net immune status rather than conventional drug exposure alone.
Doberer et al., 2025 [27]	Contemporary review/expert synthesis	Discussed TTV for immunologic risk stratification after kidney transplantation and proposed investigational interpretive ranges.	Summarizes the clinical promise of TTV while underlining the need for threshold validation and interventional evidence.

Note. The table is intended to provide a structured overview of the principal publications discussed in this narrative review and should not be interpreted as a systematic evidence table. The included articles were selected to illustrate the main clinical, analytical, and methodological aspects of TTV monitoring in kidney transplant recipients.

## Data Availability

No new data were created or analyzed in this study.
